# Combining Motion Compensation with Spatiotemporal Constraint for Video Deblurring

**DOI:** 10.3390/s18061774

**Published:** 2018-06-01

**Authors:** Jing Li, Weiguo Gong, Weihong Li

**Affiliations:** Key Lab of Optoelectronic Technology & Systems of Education Ministry, Chongqing University, Chongqing 400044, China; jinglicqu@cqu.edu.cn (J.L.); wehongli@cqu.edu.cn (W.L.)

**Keywords:** motion compensation, spatiotemporal constraint, video deblurring, blur kernel estimation

## Abstract

We propose a video deblurring method by combining motion compensation with spatiotemporal constraint for restoring blurry video caused by camera shake. The proposed method makes effective full use of the spatiotemporal information not only in the blur kernel estimation, but also in the latent sharp frame restoration. Firstly, we estimate a motion vector between the current and the previous blurred frames, and introduce the estimated motion vector for deriving the motion-compensated frame with the previous restored frame. Secondly, we proposed a blur kernel estimation strategy by applying the derived motion-compensated frame to an improved regularization model for improving the quality of the estimated blur kernel and reducing the processing time. Thirdly, we propose a spatiotemporal constraint algorithm that can not only enhance temporal consistency, but also suppress noise and ringing artifacts of the deblurred video through introducing a temporal regularization term. Finally, we extend Fast Total Variation de-convolution (FTVd) for solving the minimization problem of the proposed spatiotemporal constraint energy function. Extensive experiments demonstrate that the proposed method achieve the state-of-the-art results either in subjective vision or objective evaluation.

## 1. Introduction

The videos captured by hand-hold cameras often suffer from inevitable blur because of camera shake. As it is easy to generate global motion blur when using a tracking shot, and this type of blur widely exists in the field of mobile video surveillance, how to deblur the uniform motion blurred videos is a problem worth studying. In general, a video frame with camera shake can be modeled by a motion blur kernel, which can describes the motion blur of each video frame captured by camera in the assumption that the motion blur of each video frame is shift-invariant. Mathematically, the relationship between an observed blurry video frame and the latent sharp frame can be modeled according as follows:

(1) B=k∗L+N,

where *B*, *k*, *L* and *N* denote the observed blurry video frame, the blur kernel, the latent sharp frame and additive noise, respectively, and ∗ is convolution operator. The objective of video motion deblurring is to obtain *L* from *B*, and the problem can be converted into a blind deconvolution operation while the blur kernel is unknown.

A straightforward idea for this problem is to apply existing single or multiple image deblurring methods to each blurry frame [1,2,3,4,5,6,7,8,9,10,11,12,13,14,15]. For now, there are many mature single image deblurring methods [1,2,3,4,5,6,7,8]. Xiong et al. [1] deblurred sparsity-constrained blind image by alternating direction optimization methods. Fergus et al. [2] showed that it is possible to deblur real-world images under a sparse blur kernel prior and a mixture-of-Gaussian prior on the image gradients, but it takes a relatively long time to estimate a blur kernel for the estimation process performed in a coarse-to-fine fashion. Shan et al. [3] formulated the image deblurring problem as a Maximum a Posteriori (MAP) problem and solved it by an iterative method. A hallmark of this method is that it constrains the spatial distribution of noise by high-order models to estimate highly accurate blur kernel and latent image. Cho and Lee [4] proposed a deblurring method by introducing fast Fourier transforms (FFTs) for latent sharp frame restoration model deconvolution and using image derivatives to accelerate the blur kernel estimation, but their deblurring results are relatively sensitive to the parameters. Xu and Jia [5] proposed a texture-removal method to guide edge selection and detect large-scale structures. However, the method may fail when there are strong and complex textures in images. Krishnan et al. [6] used a L1/L2 regularization scheme to overcome the shortcomings of existing priors in an MAP setting, but it suppressed image details in the early stage during optimization. Zhang et al. [7] proposed a nonlocal blur kernel regression (NL-KR) model that exploits both the nonlocal self-similarity and local structural regularity properties in natural images, but this method is computationally expensive. Focusing on the various types of blur caused by camera shake, Kim and Lee [8] proposed an efficient dynamic scene deblurring method that does not require accurate motion segmentation with the aid of total variation (TV)-L1 based model. However, this method is not good at global motion blur.

Considering that more information will be conducive to the deblurring process, some other methods make the deblurring problem more tractable by leveraging additional input and joint multiple blurry images [9,10,11,12,13,14,15,16,17,18,19,20] in video recovery using block match multi-frame motion estimation based on single pixel cameras [9]. Tan et al. compared a blurry patch directly against the sharp candidates in spatial domain, in which the nearest neighbor matches could be recovered [10]. However, the blurry regions and the sharp regions in a frame are difficult to divide accurately in airspace. The difference of these methods is that while [11] leveraged the information in two or multiple motion blurred images, [12,13,14] employed a blurred and noisy image pair. Blurry frame could also be indicated by the inter-frame multiple images accumulation [15,16,17,18]. Tai et al. proposed a projective motion blur model with a sequence of transformation matrices [15]. Blurry images were formulated as an integration of some clear intermediate images after an optical-based transform [16]. Cho et al. proposed an approximate blur model to estimate blur function of video frames [17]. Zhang and Yao proposed a removing video blur approach that could handle non-uniform blur with non-rigid inter-frame motions [18]. However, the inter-frame multiple images accumulation model needs long program run times, because it must calculate a lot of inter-frame multiple images for estimating a blurred frame. Besides, Zhang et al. [19] described a unified multi-image deconvolution method for restoring a latent image from a given set of blurry and/or noisy observations. These multi-image deblurring methods require multiple degenerate observations of the same scene, which restricts their application in general videos. Cai et al. [20] developed a robust numerical method for restoring a sharp image from multiple motion blurred images. This method could be extended to the applicability of motion deblurring on videos, because it does not require a prior parametric model on the motion blur kernel or an accurate image alignment among frames. Nonetheless, it assumes that the input multiple images share a uniform blur kernel.

Because the temporal information of video is ignored and only the spatial prior information of an image is utilized, the performance of both single and multiple image methods is unsatisfactory while applying them to restore videos. The phenomena of artifacts, noise and inconsistencies often can be seen in the restored videos. In order to solve these problems, several video deblurring methods are explored in recent years. Takeda et al. [21] and Chan et al. [22] treated a video as a space-time volume. These methods give good spatiotemporal consistent results, however they are time-consuming as the size of space-time volume is large, and it assumes the exposure time is known in [21] and the blur kernel is identical for all frames in [22]. Qiao et al. presented a PatchMatch-based search strategy to search for a sharp superpixel to replace a blurry region [23], but each sharp superpixel was selected from a frame, so when a region in all the adjacent frames is not sharp enough, the method cannot restore the blurred region. Building upon the observation that the same objective may appear sharp on some frames whereas blurry on others, Cho et al. [17] proposed a patch-based synthesis method which ensures that the deblurred frames are both spatially and temporally coherent, because it can take full advantage of inter-frame information, but this method may fail when the camera motion is constantly large or has no sharp patches available. Besides, for solving complex motion blur, many optical flow depended methods was proposed. Wulff and Black [24] addressed the deblurring problem with a layered model and focused on estimating the parameters for both foreground and background motions with optical flow. Kim and Lee [25] proposed a method for tackling the problem by simultaneously estimating the optical flow and latent sharp frame. These methods both have strong requirements of processing time and memory consumption. To accelerate the processing, inspiring by the Fourier deblurring fusion introduced in [26,27], Delbracio and Sapiro [28] proposed an efficient deblurring method by locally fusing the consistent information of nearby frames in the Fourier domain. It makes the computation of optical flow more robust by subsampling and computing at a coarser scale. However, the method cannot effective deblurring videos with no sharp frames.

Other methods take into account the temporal coherence between video frames in the blur kernel estimation or the latent sharp frame restoration [29,30,31,32,33]. Lee et al. [29,30] utilized the high-resolution information of adjacent unblurred frames to reconstruct blurry frames. This method can accelerate the precise estimation of the blur kernel, but meanwhile it assumes that the video is sparsely blurred. Chan and Nguyen [31] introduced a L2-norm regularization function along the temporal direction to avoid flickering artifacts for LCD motion blur problem. Gong et al. [32] proposed a temporal cubic rhombic mask technique for deconvolution to enhance the temporal consistency. However, it cannot lead to sharp result because the frames used in the temporal mask term are the blurry frames adjacent to current frame, which denotes that the restoration will be close to the blurry frame. Zhang et al. [33] proposed a video deblurring approach by estimating a bundle of kernels and applying the residual deconvolution. This method has spatiotemporal consistent, but the processing time is long for estimating a bundle of kernels and iterating the residual deconvolution.

In order to solve the above-mentioned problems, we proposed a removing camera shake method for restoring the blurred videos with no sharp frames. In the proposed method, except for the spatial information, we also make full use of the temporal information for both blur kernel estimation and latent sharp frame restoration considering that the temporal information between neighboring frames can accelerate the precise estimation of blur kernel, suppress the ringing artifacts and maintain the temporal consistency of restoration. We derive a motion-compensated frame by performing motion estimation and compensation on two adjacent frames. The derived motion-compensated frame has sharp edges and little noise because it is a predictor of current sharp frame. Therefore, we apply it to a regularization model after processed for efficiently getting an accurate blur kernel. Our improved blur kernel estimation method can improve more effective restored quality than the method proposed by Lee et al. [29] in avoiding the pixel error of the motion-compensated frame and handling the blur video without sharp frame. Finally, in order to suppress the ringing artifacts and guarantee the temporal consistency in the latent sharp frame restoration step, we propose a spatiotemporal constraint term for restoring the video frames with the estimated blur kernel. The proposed spatiotemporal constraint term constrains the inter-frame information between the current sharp frame and the motion-compensated frame by the temporal regularization function rather than the temporal mask term in [32]. The proposed spatiotemporal constraint energy function is solved by extend FTVd.

The contributions of this paper can be summarized as follows:

(1) We propose a blur kernel estimation strategy by applying the derived motion-compensated frame to an improved regularization model for enhancing the quality of the estimated blur kernel and reducing the processing time.
(2) We propose a spatiotemporal constraint algorithm that introduces a temporal regularization term for obtaining latent sharp frame.
(3) We extend the computationally efficient FTVd for solving the minimization problem of the proposed spatiotemporal constraint energy function.

The rest of this paper is organized as follows: Section 2 describes the proposed method in detail. The experimental results are illustrated in Section 3. Section 4 is the conclusions.

## 2. Proposed Method

**Figure 1:**
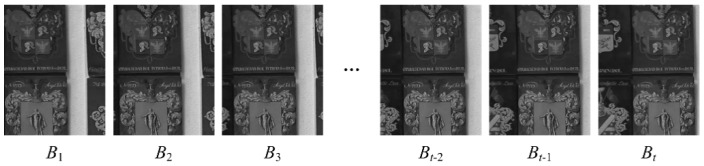
Blurry video sequence.

According to model (1), the *t*-th observed blurry frame *B*(*x*, *y*, *t*) could be related to the latent sharp frame *L*(*x*, *y*, *t*) as:

(2) B(x,y,t)=k(x,y,t)∗L(x,y,t)+N(x,y,t),

where (*x*, *y*) and *t* are the coordinate in space and time, respectively. Given a blurry video, as illustrated in Figure 1, our aim is to obtain the latent sharp frame *L* from the blurry frame *B*. Here, we focus on the uniform blur caused by camera motion, so the blur kernel is assumed to be shift-invariant. However, the blur kernel may be different from each other along the time direction, i.e., the blur kernel is spatially-invariant, meanwhile, may be temporally variant.

### 2.1. The Outline of the Proposed Video Deblurring Method

**Figure 2:**
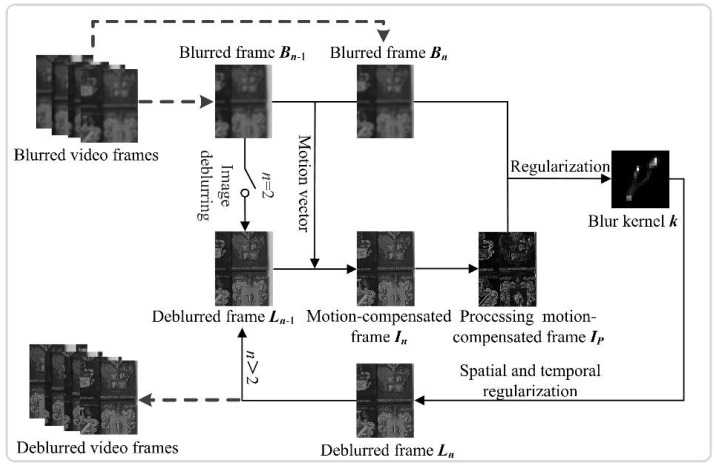
Outline of the proposed method.

A detailed description of the proposed video deblurring method is given in this section. Because there may be no sharp frame in the video, we employ a frame grouping strategy for deblurring the video. The first frame of each group is restored by a single image deblurring method, and the remaining frames of the group can be deblurred by the proposed video deblurring method with the first restored frame. For deblurring the *n*-th blurry frame in a video, our method consists of three steps and the outline of the proposed method is shown in Figure 2.

As shown in Figure 2, in the first step, we estimate the motion vector between the two adjacent blurry frames *B_n_*_−1_ and *B_n_*, and derive the motion-compensated frame *I_n_* by performing motion compensation on the previous restored frame *L_n_*_−1_. In the second step, we estimate the accurate blur kernel by the regularization algorithm with the current blurry frame *B_n_* and the preprocessed motion-compensated frame *I_P_*. In the third step, we obtain the deblurred frame *L_n_* by using the spatiotemporal constraint algorithm with the blur kernel *k* from the second step and the motion-compensated frame *I_n_* from the first step. The deblurred frame *L_n_* in the third step will be used as one of input for estimating the motion compensation and the motion-compensated frame in the next loop.

The pseudocode of the proposed video deblurring method is summarized as follows (Algorithm 1):

**Algorithm 1:** Overview of the proposed video deblurring method.**Input:** The blurry video. Divide the video into M groups that have *N* frames in a group. Set the group ordinal of the video *m* = 1 and the frame ordinal of this group *n* = 2. **Repeat** **Repeat**(1)Obtain the first deblurred frame *L*_1_ of this group by utilizing an image deblurring method.(2)Perform motion estimation algorithm to get the motion vector between the blurry frames *B_n_*_−1_ and *B_n_*, and using it to derive the motion-compensated frame *I_n_* from the previous deblurred frame *L_n_*_−1_.(3)Obtain the preprocessing motion-compensated frame *I_P_* by preprocessing *I_n_*, and then estimate the blur kernel *k* with *I_P_* and *B_n_* by the regularization method.(4)Estimate the deblurred frame *L_n_* by the spatiotemporal constraint algorithm with *k* and *I_n_*.(5)*n* ← *n* + 1.
 **Until**
*n* > *N* *m* ← *m* + 1 **Until**
*m* > *M* **Output:** The deblurred video.

### 2.2. The Proposed Blur Kernel Estimation Strategy

We first estimate the motion-compensated frame by the motion vector of the blurry frame and the previous frame for obtaining the blur kernel. We still take the *n*-th blurry frame *B_n_*, for example. Because the accuracy of the motion-compensated frame *I_n_* affects the overall performance of our method, an accurate motion vector between the current and the previous blurry frames is needed. In this paper, for generating a sufficient correct motion-compensated frame *I_n_*, according to whether the blur kernel is temporally invariant, we introduce two different matching methods that are block matching method and feature extraction method respectively.

**Figure 3:**
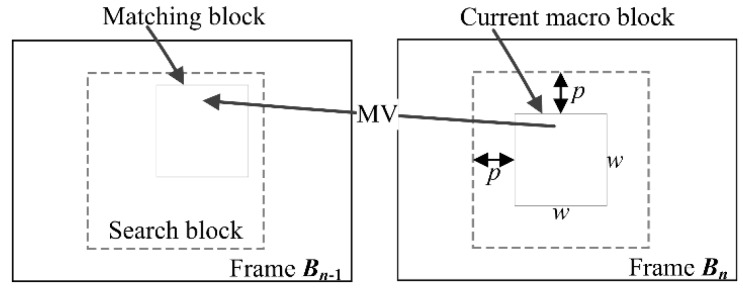
The search area of block matching algorithm.

The block matching method divides the current blurry frame into a matrix of macro block and then searches the corresponding block with the same content in the previous blurry frame. The macro block size is *w* × *w* and the searched area is constrained up to *p* pixels on all four sides of the corresponding macro block in the previous frame as shown in Figure 3. When the blur kernel is temporally invariant, all frames have exactly the same blur. As a result, an identical macro block can be found in the previous frame except the edge regions, and then a sufficiently accurate motion vector is derived. Because the exhaustive search block matching method [34] could find the best possible match amongst block matching methods, we introduce it for estimating the motion vector and set the parameters *w* = 16 and *p* = 7 as a default.

As for temporally variant blur kernels, video frames are deblurred with the blur kernels that have different sizes and directions. Consequently, we introduce a feature extraction method to track the feature points across the adjacent blurry frames due to it is robust to image blur and noise. As the Oriented Fast and Rotated BRIEF (ORB) method [35] is much faster than the other extraction methods and shows good performance on blurry images [36], we employ the method to estimate the motion vector. Firstly, we match the feature points between the adjacent frames. Then, we calculate the mean motion vector of all feature points when the scene is static for that all the pixels have a same motion vector. When there are moving objects in the scene, the frames are divided into a matrix of macro blocks, and the motion vector of each block is dependent on the feature points in the current block and its neighborhood blocks.

After obtaining the motion vector between the adjacent blurry frames *B_n_*_−1_ and *B_n_*, the motion-compensated frame *I_n_*, i.e., the initial estimation of the current sharp frame can be derived by performing motion compensation on the previous deblurred frame *L_n_*_−1_, which is estimated in the previous loop. It should be noted that the first deblurred frame *L*_1_ of each group can be achieved by an image deblurring method.

We estimate the blur kernel by edge information after obtained the motion-compensated frame. Cho and Lee [4] estimate the blur kernel by solving the energy function similar to:

(3) $$E_{k}(k)={\left\|k*I-B\right\|}^{2}+\alpha {\left\|k\right\|}^{2},$$

where ‖k∗I−B‖ is the data term, and ${\left\|\cdot \right\|}^{2}$ is L2-norm. *B* is the current blurry frame, namely, *B_n_*, *I* is the latent sharp frame, and *α* is a weight for the regularization term ${\left\|k\right\|}^{2}$.

In energy function (3), the blurry frame is used to estimate the blur kernel, the latent sharp frame *I* has to be obtained firstly through the prior information of the current frame. Considering that it takes a great deal of time if a coarse-to-fine scheme or an alternating iterative optimization scheme is employed, Cho and Lee used a simple de-convolution method to estimate the latent sharp frame *I* and formulated the optimization function using image derivatives rather than pixel values to accelerate the blur kernel estimation. However, the method needs to estimate the latent sharp frame without the inter-frame information of the video, and the estimated one is of enough sharp edges.

In order to take full advantages of the temporal information and accelerate the precise estimation of the blur kernel, we propose a blur kernel estimation strategy based on [4] which applying the motion-compensated frame *I_n_* to the data term of (3) for *I_n_* is pretty close to the current latent sharp frame. However, there may exist error of *I_n_*, and as illustrated in [4], sharp edges and noise suppression in smooth regions will enable accurate kernel estimation. For obtaining salient edges, removing noise, and avoiding the influence of the errors, we preprocess *I_n_* by anisotropic diffusion and shock filter to get a preprocessing motion-compensated frame *I_P_*.

The anisotropic diffusion equation is as follows:

(4) $$\frac{\partial I}{\partial t}=div\left(c\left(\left\|\nabla I\right\|\right)\nabla I\right),$$

where *div* and ∇ are the divergence operator and the gradient operator respectively. c(‖∇I‖) denotes the coefficient of diffusion and can be obtained by using:

(5) $$c\left(\left\|\nabla I\right\|\right)=\frac{1}{1+{\left(\left\|\nabla I\right\|/g\right)}^{2}},$$

where *g* is the gradient threshold and is set to 0.05 as a default.

The evolution equation of a shock filter is formulated as follows:

(6) $$I_{t+1}=I_{t}-sign\left(\Delta I_{t}\right)\left\|\nabla I_{t}\right\|dt,$$

where *I_t_* is an image at time *t*, Δ and ∇ are the Laplacian and gradient operators, respectively, *dt* is the time step for a single evolution and is set to 0.1 in the experiments.

The anisotropic diffusion is firstly applied to the motion-compensated frame *I_n_* and then the shock filter is used to obtain the preprocessing motion-compensated frame *I_P_*. Due to the fact the above processing steps can sharpen edges and discard small details, the motion estimation errors have little effect on the blur kernel estimation. So, an accurate blur kernel can be estimated by energy function (3), where we use the preprocessing motion-compensated frame *I_P_* as *I* in the data term. The parameter *α* is set to 1 in our experiments. Besides the proposed blur kernel estimation strategy without iterative can improve greatly the running speed.

For solving energy function (3), we perform the fast Fourier transform (FFT) on all variables and then set the derivative of *k* to 0 for solving the minimization problem. Hence, the equation of *k* is derived as follows:

(7) $$k=F^{-1}\left(\frac{\bar{F\left(I_{P}\right)}\circ F\left(B\right)}{\bar{F\left(I_{P}\right)}\circ F\left(I_{P}\right)+\alpha }\right),$$

where F and $F^{-1}$ denote the forward and inverse FFT, respectively, and $\bar{F\left(I_{P}\right)}$ is the complex conjugate of $F\left(I_{P}\right)$, ∘ is an element-wise multiplication operator.

### 2.3. The Proposed Spatiotemporal Constraint Algorithm

We propose a kind of new spatiotemporal constraint algorithm for obtaining latent sharp frame. The proposed model is improved from energy function (8) that initially is proven in [31]:

(8) $$E_{L}(L)={\left\|k*L-B\right\|}_{2}^{2}+\lambda \sum_{i}{\left\|D_{i}L\right\|}_{1}+\beta {\left\|L-ML_{0}\right\|}_{2}^{2},$$

where ${\left\|\cdot \right\|}_{1}$ is L1-norm, and *D_i_* is the spatial directional gradient operators at 0°, 45°, 90° and 135°, *L*, *L*_0_, and *M* represent the current sharp frame, the previous deblurred frame, and the motion compensation, respectively, *ML*_0_ is equivalent to the motion-compensated frame *I_n_*, *λ* and *β* are two regularization parameters.

The first part of energy function (8) is a data term, where the image pixel values are calculated. However, in the data term, the noise for all pixels cannot capture at all the spatial randomness of noise, and that would lead to deconvolution ringing artifacts. For reducing the ringing artifacts of image deconvolution, we introduce the likelihood term proposed by Shan et al. [3] as shown in the first term of energy function (9). In the latter two terms of energy function (8), the spatial regularization function employs L1-norm to suppress noises and preserve edges, and the temporal regularization function employs L2-norm to maintain the smoothness along time axis. These regularization functions are capable of reducing the spatiotemporal noise, as well as keeping the temporal coherence of the deblurred video. However, it is inevitable that a few errors exist during motion estimation and compensation. Since the temporal regularization term makes the estimated current sharp frame close to the motion-compensated frame for each image pixel, the errors of motion estimation and compensation give rise to a deviation in the estimated current sharp frame. We propose a temporal regularization constraint term with L2-norm on the differential operators that able to avoid introducing pixel errors.

**Figure 4:**
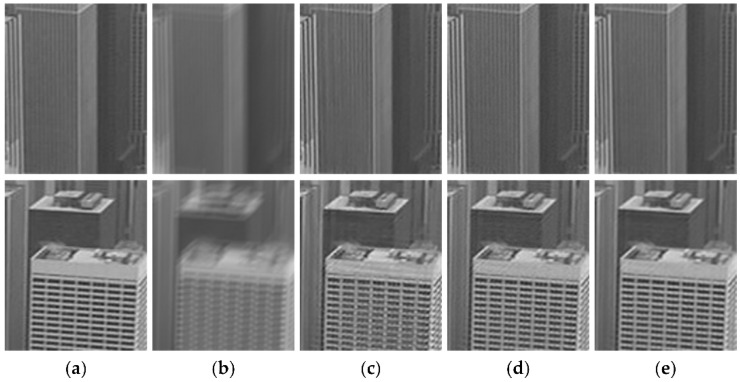
Comparison between different deconvolution algorithms. (**a**) Original input frame. (**b**) Blurred frame. (**c**) Deblurred result by minimizing $\left(\sum_{\partial^{*}}\omega_{k\left(\partial^{*}\right)}{\left\|k*\partial^{*}L-\partial^{*}B\right\|}_{2}^{2}\right)+\lambda_{S}{\left\|\nabla L\right\|}_{1}$. (**d**) Deblurred result by minimizing$\left(\sum_{\partial^{*}}\omega_{k\left(\partial^{*}\right)}{\left\|k*\partial^{*}L-\partial^{*}B\right\|}_{2}^{2}\right)+\lambda_{S}{\left\|\nabla L\right\|}_{1}+\lambda_{T}{\left\|L-L^{mc}\right\|}_{2}^{2}$. (**e**) Deblurred result by minimizing the proposed algorithm$\left(\sum_{\partial^{*}}\omega_{k\left(\partial^{*}\right)}{\left\|k*\partial^{*}L-\partial^{*}B\right\|}_{2}^{2}\right)+\lambda_{S}{\left\|\nabla L\right\|}_{1}+\lambda_{T}{\left\|\nabla L-\nabla L^{mc}\right\|}_{2}^{2}$.

For illustrating the effectiveness of the temporal regularization function, the proposed deconvolution algorithm is compared with the spatial regularization algorithm and the L2-norm temporal regularization based deconvolution algorithm as shown in Figure 4. The comparison results show that the smoothness of the restored result in Figure 4c by the spatial regularization algorithm without temporal regularization term is poor, and the restored result in Figure 4d by the L2-norm temporal regularization based deconvolution algorithm contains some noise. As shown in Figure 4e, the result restored by our algorithm has sharper edges than the above algorithms.

The proposed energy function is as follows:

(9) $$E_{L}(L)=\sum_{\partial^{*}}\omega_{k\left(\partial^{*}\right)}{\left\|k*\partial^{*}L-\partial^{*}B\right\|}_{2}^{2}+\lambda_{S}{\left\|\nabla L\right\|}_{1}+\lambda_{T}{\left\|\nabla L-\nabla L^{mc}\right\|}_{2}^{2},$$

where $\partial^{*}\in \left\{\partial_{0},\partial_{x},\partial_{y},\partial_{xx},\partial_{xy},\partial_{yy}\right\}$ stands for the partial derivative operators and $\omega_{k\left(\partial^{*}\right)}$ is a series of weights for each partial derivative, which is determined as Shan et al. [3], *λ_S_* and *λ_T_* are the spatial and temporal regularization constraint parameters respectively. When *λ_T_* is too small, the deblurred frames are not smoothness enough. When *λ_T_* is too large, the accumulated error in time axis can be amplified, especially for large loop numbers. Therefore, *λ_T_* is calculated according to the ordinal of the frame in a group. ∇ represents the first difference operator and $L^{mc}$ is the motion-compensated frame, i.e., $L^{mc}=I_{n}$.

Then, we extend FTVd for solving the minimization problem of energy function (9) effectively. Main idea of FTVd is to employ the splitting technique and translate the problem to a pair of easy subproblems. To this end, an intermediate variable *u* is introduced to transform energy function (9) into an equivalent minimizing problem as follows:

(10) $$E_{L}(L)=\left(\sum_{\partial^{*}}\omega_{k\left(\partial^{*}\right)}{\left\|k*\partial^{*}L-\partial^{*}B\right\|}_{2}^{2}\right)+\lambda_{S}{\left\|u\right\|}_{1}+\lambda_{T}{\left\|\nabla L-\nabla L^{mc}\right\|}_{2}^{2}+\gamma {\left\|u-\nabla L\right\|}_{2}^{2},$$

where *γ* is a penalty parameter, which controls the weight of the penalty term ${\left\|u-\nabla L\right\|}_{2}^{2}$.

Next, we solve problem (10) by minimizing the following subproblems:

*u*-Subproblem: With *L* fixed, we update *u* by minimizing:

(11) $$E_{u}^{\prime }(u)=\frac{\lambda_{S}}{\gamma }{\left\|u\right\|}_{1}+{\left\|u-\nabla L\right\|}_{2}^{2}.$$

Using the shrinkage formula to solve this problem, *u_x_* and *u_y_* are given as follows:

(12) $$u_{x}=\max\left(\left|\partial_{x}L\right|-\frac{\lambda_{S}}{\gamma },0\right)\cdot \mathrm{sign}\left(\partial_{x}L\right).$$

(13) $$u_{y}=\max\left(\left|\partial_{y}L\right|-\frac{\lambda_{S}}{\gamma },0\right)\cdot \mathrm{sign}\left(\partial_{y}L\right).$$

*L*-subproblem: By fixing *u*, (10) can be simplified to:

(14) $$E_{L}^{\prime }=\left(\sum_{\partial^{*}}\omega_{k\left(\partial^{*}\right)}{\left\|k*\partial^{*}L-\partial^{*}B\right\|}_{2}^{2}\right)+\lambda_{T}{\left\|\nabla L-\nabla L^{mc}\right\|}_{2}^{2}+\gamma {\left\|u-\nabla L\right\|}_{2}^{2}.$$

The blur kernel *k* is a block-circulant matrix. Hence, (14) has the following solution according to Plancherel’s theorem:

(15) $$L^{*}=F^{-1}\left(\frac{\bar{F\left(k\right)}\circ F\left(B\right)\circ \Delta_{1}+\lambda_{T}F\left(L^{mc}\right)\circ \Delta_{2}+\gamma \left(\bar{F\left(\partial_{x}\right)}\circ F\left(u_{x}\right)+\bar{F\left(\partial_{y}\right)}\circ F\left(u_{y}\right)\right)}{\bar{F\left(k\right)}\circ F\left(k\right)\circ \Delta_{1}+\left(\gamma +\lambda_{T}\right)\Delta_{2}}\right),$$

where $\Delta_{1}=\sum_{\partial^{*}}\omega_{K\left(\partial^{*}\right)}\bar{F\left(\partial^{*}\right)}\circ F\left(\partial^{*}\right)$ and $\Delta_{2}=\bar{F\left(\partial_{x}\right)}\circ F\left(\partial_{x}\right)+\bar{F\left(\partial_{y}\right)}\circ F\left(\partial_{y}\right)$.

Algorithm 2 is the pseudocode of the proposed spatiotemporal constraint algorithm.

**Algorithm 2:** The proposed spatiotemporal constraint algorithm.**Input:** the blurry frame *B_n_* (n≥2), the motion-compensated frame *I_n_*, the blur kernel *k* and the parameters *λ_S_* and *λ_T_*. Initialize the deblurred frame *L* = *B_n_*. **While** not converge **do**(1)Save the previous iterate: *L_p_* = *L*.(2)With *L* fixed, solve the *u*-subproblem using (12) and (13).(3)With *u* fixed, solve the *L*-subproblem using (15).
   **If**
${\left\|L-L_{P}\right\|}_{2}/{\left\|L_{P}\right\|}_{2}\leq tol$
**then**   Break   **End if** **End while** **Output:** the deblurred frame L.

## 3. Experimental Results and Discussion

### 3.1. Experimental Settings

In order to demonstrate the effectiveness of the proposed method, some artificially and naturally uniform blurred videos are implemented to make a series of experiments. We also perform comparison with the several representative image and video deblurring methods, such as Shan’s method [3], Cho’s method [4], Chan’s method [22], Cho’s method [17], Kim’s method [25], Lee’s method [29] and Gong’s method [32]. The performance of these methods are measured by the visual and objective evaluation, the latter includes the increase in signal to noise ratio (ISNR) [37] and peak signal to noise ratio (PSNR) [38]. In the following comparison experiments, the images and codes are provided by the authors, and the parameters are hand-tuned to produce the best possible results according to corresponding papers. All experiments conducted in the MATLAB 2016a environment on a desktop PC equipped with a 3.20 GHz Intel Core Xeon CPU and 3.48 GB memory. In our experiments, we set *N* = 8, *α* = 1, the parameters *λ_S_* and *λ_T_* are set to 1/*mu* and 5/[*mu*(*n* − 1)], respectively, where *mu* is set to the experience value 120 and n is the ordinal of the frame in a group. The penalty parameter *γ* is set to *β*_2_/*mu*, where *β*_2_ is set to the experience values 100.

### 3.2. Artificially Blurred Videos

**Figure 5:**
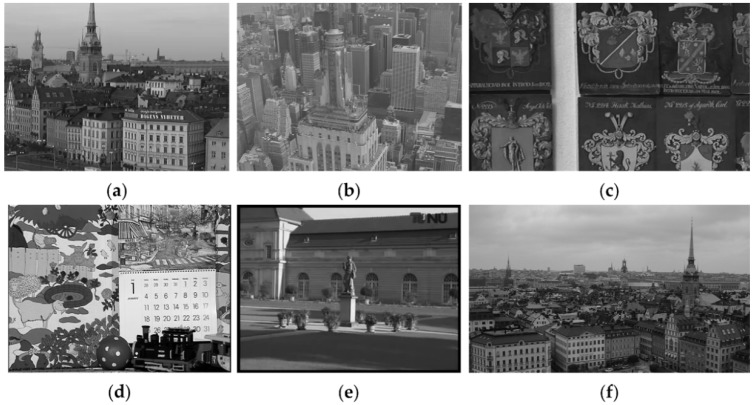
Videos for artificially blurred experiments. (**a**–**f**) are respectively videos *stockholm*, *city*, *shield*, *mobile* & *calendar*, *tu berlin* and *old town cross*.

**Figure 6:**
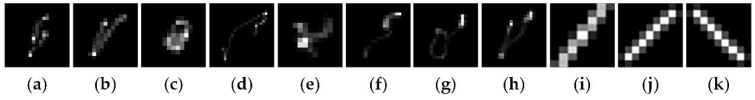
Blur kernels for artificially blurred experiments. (**a**–**k**) Sizes of 17 × 17, 15 × 15, 13 × 13, 25 × 25, 11 × 11, 19 × 19, 21 × 21, 21 × 21, 7 × 9, 9 × 9 and 9 × 9, respectively.

For verifying the effectiveness of the proposed method when restoring artificially blurred videos, we perform comparative experiments on six grayscale videos with several motion types and the results are shown in Figure 5, where the cameras which capture the videos *stockholm* and *shield* are quite similar and undergo translational motion and that which captures the video *old town cross* has depth variance motion. The videos *city* and *tu berlin* include both translational and rotation motion, but the former has more details. The video *mobile* & *calendar* is a dynamic scene, whose blur is caused by the camera with depth variance motion and the objects with complex motion. The above videos are artificially blurred by the different methods with the blur kernels as shown in Figure 6. The first method is the temporally variant artificially blur method that the frames of a video are convoluted with different complex blur kernels. The second method is that all frame of a video are convoluted with a same linear blur kernel. Figure 6a–h shows the complex blur kernels which are provided from [39] for generating the temporally variant blur video. The blur kernels are generated by camera motion on a tripod. The Z-axis rotation handle of the tripod is locked and the X-axis and the Y-axis handles are loosened. The camera is set as an 85 mm lens and a 0.3 s exposure. The other three blur kernels as shown in Figure 6i–k is the linear blur kernels for generating the temporally invariant blur video. The direction of the three blur kernels are 60, 45 and 135, respectively. In addition, we add the Gaussian noise with standard variance as 0.001 to the blurred frames. In order to avoid the negative influence of the single image deblurring method, we assume that the first latent sharp frame is known in subsequent experiments, which should be obtained by the image deblurring method in reality.

#### 3.2.1. Temporally Invariant Blur Kernel

**Figure 7:**
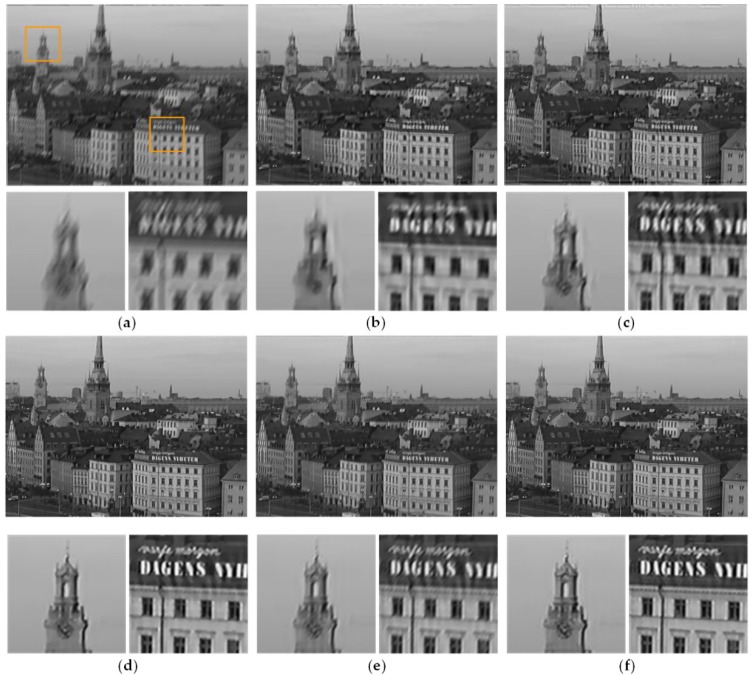
Deblurred results of video *Stockholm* by different methods. (**a**) Original blurry frame. (**b**) Method [3]. (**c**) Method [4]. (**d**) Method [22]. (**e**) Method [32]. (**f**) Ours.

**Figure 8:**
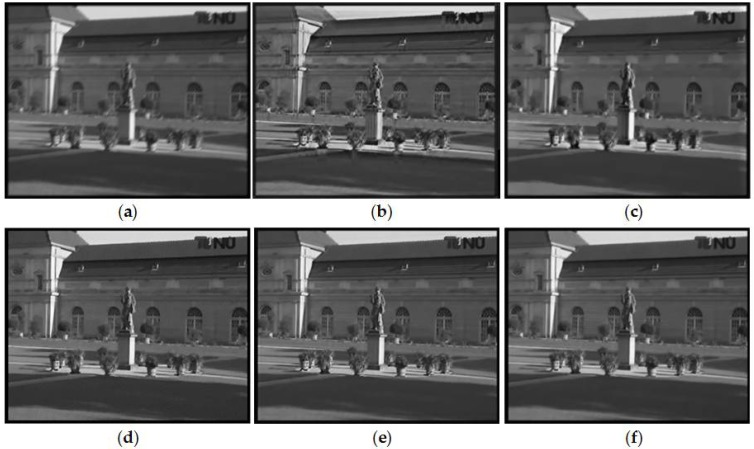
Deblurred results of video *Tu berlin* by different methods. (**a**) Original blurry frame. (**b**) Method [3]. (**c**) Method [4]. (**d**) Method [22]. (**e**) Method [32]. (**f**) Ours.

**Figure 9:**
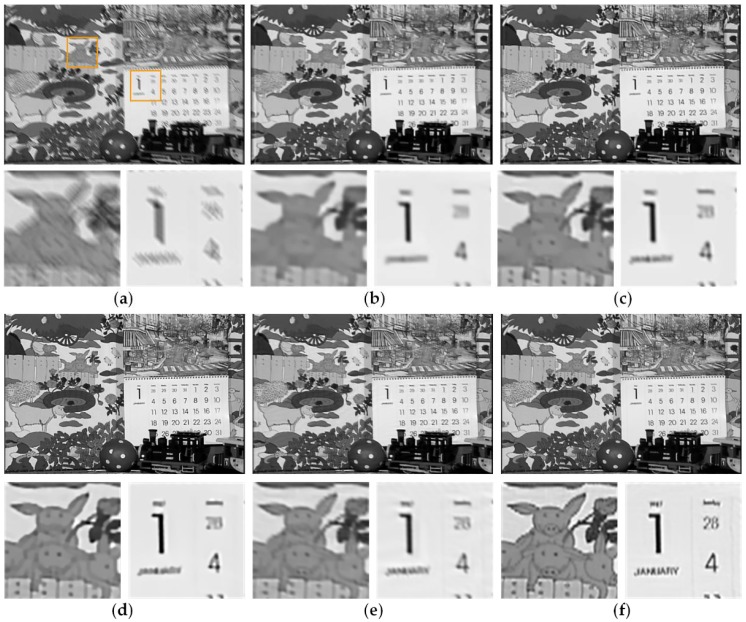
Deblurred results of video *Mobile & calendar* by different methods. (**a**) Original blurry frame. (**b**) Method [3]. (**c**) Method [4]. (**d**) Method [22]. (**e**) Method [32]. (**f**) Ours.

We first consider the class of temporally invariant blur, which assumes that the blur kernels are identical for all frames. Thus, the exhaustive search block matching method is utilized for motion estimation. The artificially blurred videos are generated by the same linear blur kernel convolute the all frames of a video. We test the proposed method on three sample videos with the different linear blur kernels in Figure 6i–k, respectively. The comparative experiment of method [22], which is a non-blind deblurring method, uses the same blur kernel of our method. Figure 7, Figure 8 and Figure 9 show the comparison results between our method and methods [3,4,22,32]. In the partial enlarged images of Figure 7 and Figure 9, there are many ripples around the edges in the deblurred frames by methods [3] and [4]. The deblurred frames by method [22] lose many details. The deblurred frames by method [32] are sharper than that by the above methods, but they still contain somewhat artifacts. In contrast, the deblurred frames by our method contain more small-scale details and fewer artifacts.

For illustrating the accuracy of the improved blur kernel estimation method, the blur kernels of Figure 7, Figure 8 and Figure 9 are evaluated by an objective evaluation. Table 1 shows the errors of the estimated blur kernels by different methods, which are measured by the sum of pixel-wise squared differences between the estimated blur kernels and original blur kernels. In the following Tables, the rough font represents the best result.

The blur kernels by our method have the least errors in videos *stockholm* and *tu berlin*. In video *mobile* & *calendar*, the accuracy of our method is similar to method [4]. Table 2 shows the average ISNR results and the processing times by the different methods. We compared the computational complexities of our method and other methods by the processing time of restoring blurred video. Since we employ a grouping strategy and assume the first frame of each group is sharp, we calculate the average ISNR of each group except for the first frame. Meanwhile, the average ISNR of method [22] also is calculated because it adopts the same blur kernel as our method for each frame. The highest ISNR values and the least processing time of the three videos all are calculated by our method, except for method [4].

The six videos in Figure 5 are artificially blurred with the blur kernels in Figure 6. The average PSNR results are compared among the different methods in Table 3. The comparison results indicate that our method has the highest PSNR.

Method [29] uses a similar blur kernel estimation strategy to our method, hence our method is compared with method [29]. Firstly, 20 frames are extracted from video *shields* and *city* randomly. Then, these frames are blurred with the blur kernels in Figure 6, respectively. Table 4 shows the mean and variance of the ISNR results for the deblurred videos *shields* and *city*. From Table 4, we can see that our method has the higher mean as well as the lower variance compared with method [29].

#### 3.2.2. Temporally Variant Blur Kernel

**Figure 10:**
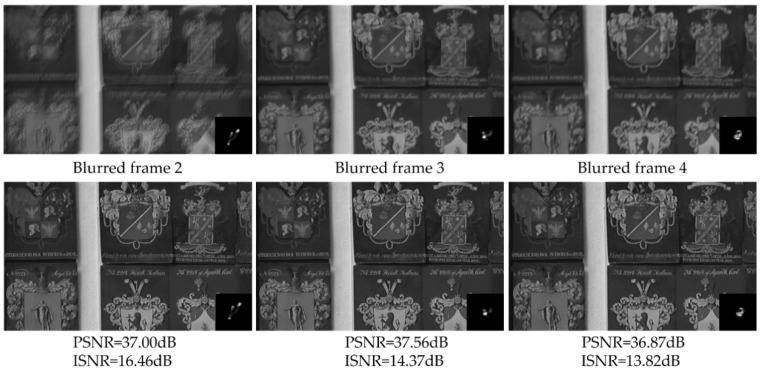
Three consecutive video sequences of video *shield*. Top: the artificially blurred video sequences with different blur kernels. Bottom: the corresponding deblurred frames by our method.

The proposed method can be used to remove temporally variant motion blur as shown in Figure 10. In the circumstance, because the blur kernel of each frame may be different, the motion vectors are estimated by the ORB method. The top of Figure 10 shows three consecutive artificially blurred video sequences which are blurred with the frames 2 to 4 from video *shield* and the random blur kernels, such as Figure 6c,e,h. The bottom of Figure 10 is the corresponding deblurred frames by our method, which have sharp edges and visible details, as well as high PSNR and ISNR values.

### 3.3. Naturally Blurry Videos

**Figure 11:**
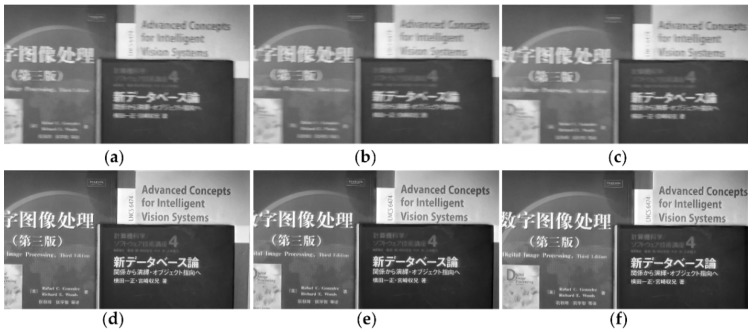
Naturally blurry video sequence *book*. (**a**–**c**) are the continuous blurry video sequences. (**d**–**f**) are the corresponding deblurred frames of (**a**–**c**) by our method.

In addition to some artificially blurred videos, we also apply the proposed method to naturally blurry videos to further demonstrate the effectiveness of our method. Figure 11 shows the deblurred results of the naturally blurry video by our method. Figure 11a–c is the consecutive three frames of the naturally blurry video *book* which are captured by a SONY HDR-PJ510E hand-held camera with translation motion in the horizontal direction and slight camera rotation. The original color videos are transformed into grayscale. Figure 11d–f is the corresponding deblurred frames of Figure 11a–c by our method. Since we assume that there is no sharp frame in the blurred video, an image deblurring method should be adopted to restore the first frame. Here, we use method [32] to estimate the blur kernel and use the latent sharp frame restoration method without temporal cubic rhombic mask to restore the first frame. Then we utilize the proposed spatiotemporal frame correlation method to deblur the remaining frames in the group.

**Figure 12:**
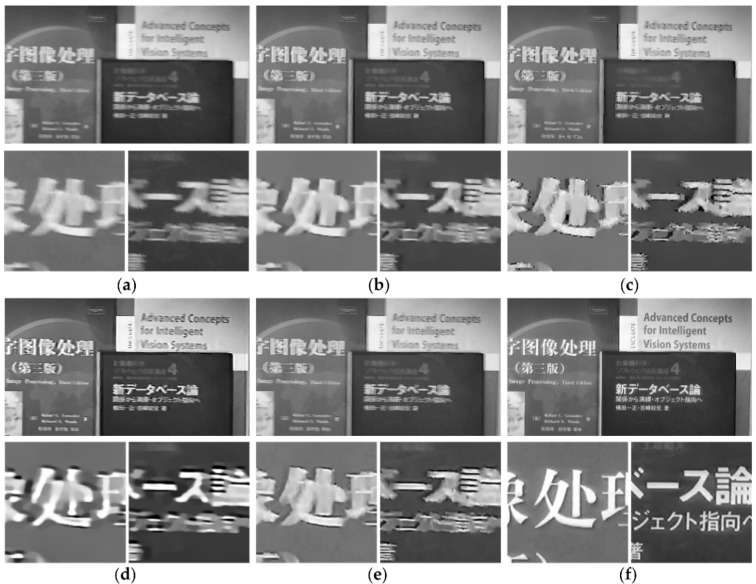
Naturally blurry video *book*. (**a**) *Original* blurry frame. (**b**–**f**) Deblurred results by different methods. (**b**) Method [3]. (**c**) Method [4]. (**d**) Method [22]. (**e**) Method [32]. (**f**) Ours.

Figure 12 shows the deblurred results of naturally blurry video *book* by the different methods. Figure 12a is a naturally blurry frame which is randomly chosen from video *book*, such as the frame in Figure 11a. In Figure 12, the frames deblurred by method [3,4] contain noticeable ringing artifacts while that obtained by method [22] presents massive blocky deformation and loses many details. The deblurring result by method [32] is relatively better than the above methods, but there are multiple ringing artifacts in the object edges, whereas, the results deblurred by our method have sharper edges and better local details than those obtained by the other methods.

**Figure 13:**
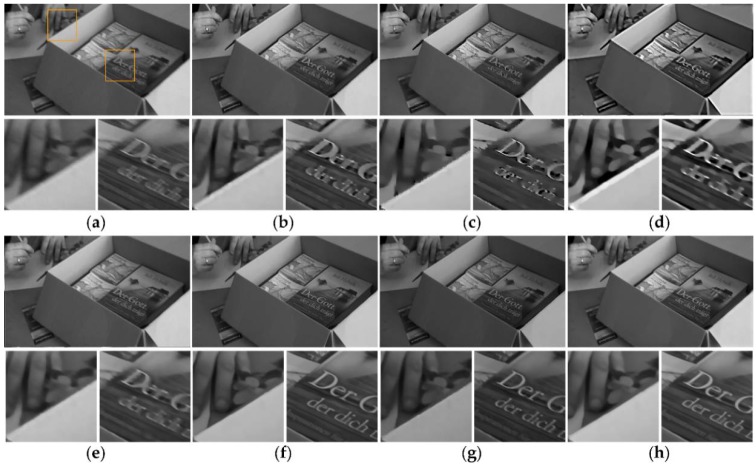
Naturally blurry video *books*. (**a**) Original blurry frame. (**b**–**h**) Deblurred results by different methods. (**b**) Method [3]. (**c**) Method [4]. (**d**) Method [22]. (**e**) Method [32]. (**f**) Method [17]. (**g**) Method [25]. (**h**) Ours.

**Figure 14:**
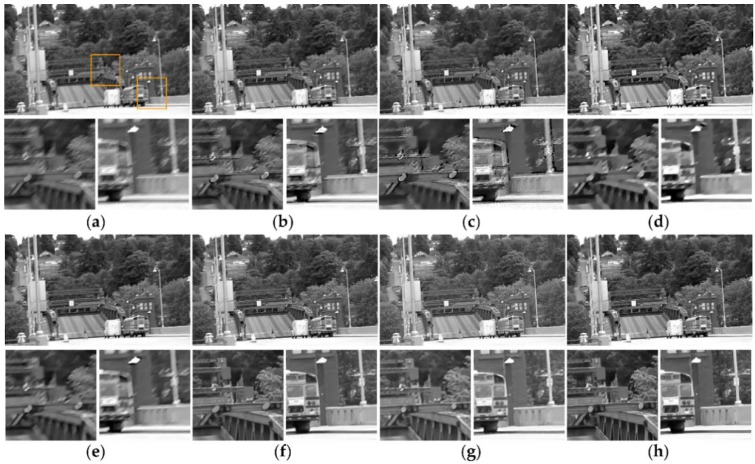
Naturally blurry video *bridge*. (**a**) Original blurry frame. (**b**–**h**) Deblurred results by different methods. (**b**) Method [3]. (**c**) Method [4]. (**d**) Method [22]. (**e**) Method [32]. (**f**) Method [17]. (**g**) Method [25]. (**h**) Ours.

The widely used videos *books* and *bridge* provided by Cho et al. [17] are used to perform naturally blurry video experiments. Because we focus on handling the blur frame that are captured by translational camera, several frames of videos *books* and *bridge* which have uniform motion blur are chosen to make the comparison experiments. Our method is compared not only with the previous uniform image and video deblurring methods, but also with the patch-based method [17] and the optical flow method [25]. The experimental results are shown in Figure 13 and Figure 14.

In Figure 14b,c, the frames deblurred by methods [3,4] contain noticeable ringing artifacts and burrs. The deblurred frames by method [22,32] present massive blocky deformation and lose many details. In Figure 14f, method [17] fails to deblur the region around the traffic lights. Method [17] cannot properly match a sharp patch with a burry one in the presence of saturated pixels. Moreover, this method would fail when the frames are constantly blur since it needs to find sharp patches. As illustrated in Figure 13 and Figure 14, our method obtains better or similar results than method [17]. The general video deblurring method [25] also produces relatively good quality results. However, the over-smoothing phenomenon can be observed in Figure 14g, where many details are lost, whereas, our method produces a relatively reasonable deblurred result with significantly sharper edges and more visible details than the other methods. In addition, method [25], which calculates the blur kernel of each pixel, requires huge storage space and long processing time.

For objectively evaluating the accuracy of the proposed video deblurring method on naturally blurry video, a no-reference sharpness metric base on the local gradients distribution to quantify the blur amount [33] is used to evaluate the deblurred results by different methods. The no-reference sharpness metric estimated method is that divided the larger one of the two singular values of the gradient matrix at each pixel by the number of the pixels at a frame. A bigger sharpness value indicates more sharp appearance of the frame.

Table 5 shows the average no-reference sharpness metric results of the naturally blurry videos *book*, *books* and *bridge* by different methods. From Table 5, we can see that the no-reference sharpness metric results of our method are higher than other methods except method [22]. However, the deblurred frames by method [22] are significant deformation as show in Figure 13d and Figure 14d.

### 3.4. Effects of the Restored First Frame and the Motion-Compensated Frame

**Figure 15:**
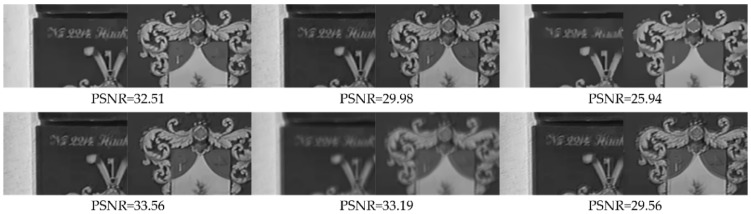
The effect of the restored first frame on the deblurred frame. Top: The restored first frames with different accuracies. Bottom: The corresponding deblurred second frames by our method.

Effects of the restored first frame and the motion-compensated frame on the final restored results are evaluated in objective evaluation and subjective vision respectively. In order to illustrate the effect of the restored first frame on the final restored results, the randomly adjacent frames of the video *mobile* & *calendar* are artificially blurred with the blur kernel shown in Figure 6e. For obtaining the restored first frames with different accuracies, the artificially blurred first frame is restored by the image deblurring method [4] with different parameters, respectively. Then, for comparison, the second frames are deblurred by our method with the restored first frames with different accuracies, respectively. Figure 15 shows the experiment result, the first frame of top is the original first frame before artificially blur and the latter two frames of top are the restored first frames with different accuracies. These three frames respectively as the restored first frame input to the second frame deblurring process, and the corresponding deblurred results for the second frame by our method are shown in bottom of Figure 15. The PSNR results in Figure 15 demonstrate that the deblurred results by our method are not sensitive to the accuracy of the restored first frame when the restored first frame has sharp enough edges. The robustness owes to the processing method, and the sharper restored first frame is, the better deblurred results are.

**Figure 16:**
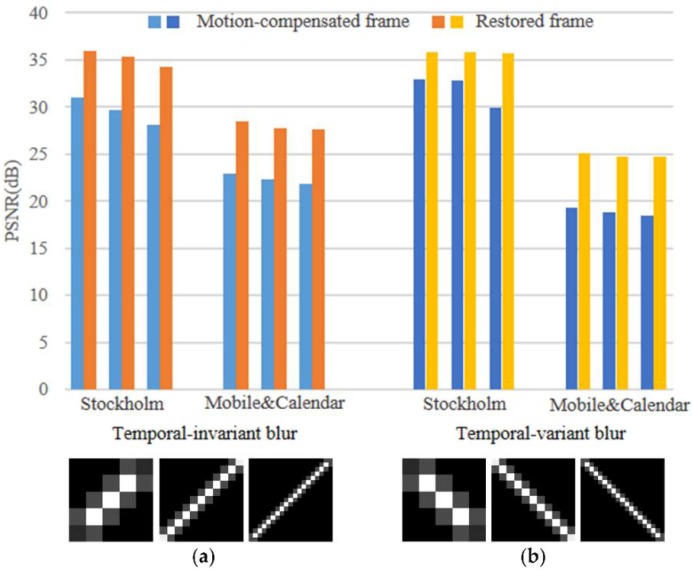
Effect of the motion-compensated frame on the restored frame by our method. Top: The PSNR results of the motion-compensated frames and the restored frames. Bottom: The blur kernels used for degradation.

**Figure 17:**
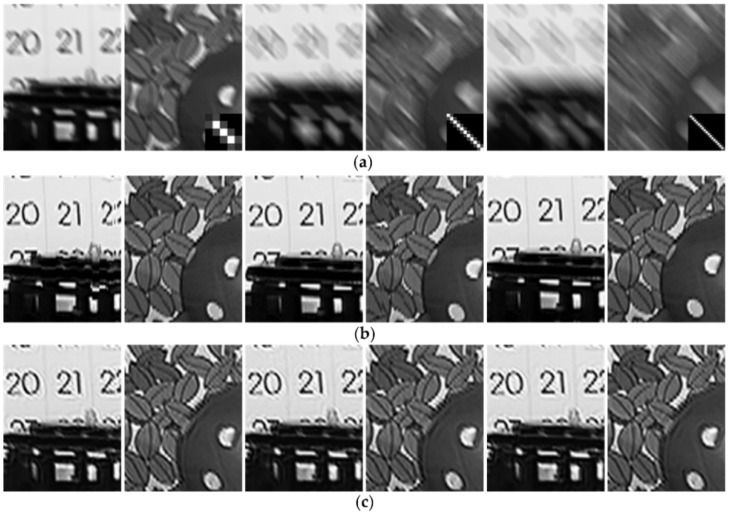
The motion-compensated frame and the restored frames by our method. (**a**) The artificially blurred second frames with the blur kernels of Figure 16b. (**b**,**c**) are the corresponding restored frames of (**a**) in the situation of temporal-invariant and temporal-variant blur, respectively.

To illustrate the influence of the motion-compensated frame on the final restored result, we execute the experiments on the static and dynamic scene videos *stockholm* and *mobile* & *calendar*, respectively. For the temporal-invariant blur, the blur kernels used for degradation are linear motion blurs, such as Figure 16b. For the temporal-variant blur, the first frames are blurred by the blur kernels as Figure 16a and the corresponding second frames are blurred by the blur kernels as Figure 16b. Figure 16a,b are the blur kernels of the 45 and 135 degree directions, and the blur kernels sizes are 5 pixels, 15 pixels, and 25 pixels, which correspond to mild blur, moderate blur and severe blur, respectively. The top of Figure 16 shows the PSNR results of the motion-compensated frame and the restored frame for the artificially blurred second frame by our method. The artificially blurred second frames and the corresponding restored frames are shown in Figure 17. Comparing the results as shown in Figure 16 and Figure 17, due to our method has great robustness to the error of motion estimation and compensation, the restored frames have sharp edges and visible details, and the PSNR results of the restored frames are significantly higher than that of the motion-compensated frames.

## 4. Conclusions

In this paper, we proposed a video deblurring method by combining motion compensation with spatiotemporal constraint. A blur kernel estimation strategy is proposed by applying the derived motion-compensated frame to an improved regularization model for enhancing the quality of the estimated blur kernel and reducing the processing time. We also proposed a spatiotemporal constraint algorithm which introduces a temporal regularization term for obtaining the latent sharp frame. We extend FTVd for solving the minimization problem of the proposed spatiotemporal constraint energy function. Because it makes effective use of the relationship between the current frame and the motion-compensated frames, our method can more accurately estimate the blur kernel without expensive computation, and more effectively suppress the ringing artifacts and maintain the spatiotemporal consistencies of the deblurred video.

The artificially and naturally experimental results illustrated that no matter whether the blur kernel is temporally variant or not, our method could effectively restore the latent sharp frame with details and without noticeable artifacts. Moreover, the quantitative comparison results on a publicly available datasets demonstrated that the proposed method surpass the state-of-the-art methods.

## Figures and Tables

**Table 1:** Errors (10^−3^) results of the estimated blur kernels by different methods.

Methods	Videos		
	*Stockholm*	*Tu Berlin*	*Mobile* & *Calndar*
[3]	8.91	16.01	0.38
[4]	3.78	18.02	**0.23**
[32]	3.32	2.72	0.32
Ours	**1.99**	**1.56**	0.25

**Table 2:** Average ISNR (dB) results and processing timses (sec) by different methods.

Methods	ISNR			Processing Time			Language
	*Stockholm*	*Tu Berlin*	*Mobile* & *Calendar*	*Stockholm*	*Tu Berlin*	*Mobile* & *Calendar*	
[3]	4.32	2.72	5.01	68.34	56.16	73.14	C++
[4]	6.46	5.68	6.90	**3.20**	**1.10**	**3.50**	C++
[22]	9.28	9.52	7.73	-	-	-	-
[32]	9.61	9.48	8.54	33.56	15.54	37.27	MATLAB
Ours	**11.14**	**13.31**	**9.01**	8.81	1.91	10.43	MATLAB

**Table 3:** Average PSNR (dB) results of the artificially blurred videos.

Methods	Videos					
	*Stockholm*	*Shield*	*Mobile* & *Calendar*	*City*	*Tu Berlin*	*Old Town Cross*
[3]	26.28	28.43	21.39	27.54	24.29	28.32
[4]	28.53	30.07	24.32	26.53	23.63	30.03
[22]	31.76	31.55	26.30	28.76	33.32	32.25
[32]	28.24	28.48	22.88	26.61	28.54	30.12
Ours	**33.92**	**33.53**	**26.36**	**30.54**	**35.55**	**34.23**

**Table 4:** Comparison with method [29] for videos *shields* and *city*.

Methods	Mean		Variance	
	*Shields*	*City*	*Shields*	*City*
[29]	9.71	9.66	1.35	0.37
Ours	**12.04**	**10.62**	**1.10**	**0.21**

**Table 5:** Average no-reference sharpness metric results of the naturally blurry videos by different methods.

Methods	No-Reference Sharpness Metric		
	*Book*	*Books*	*Bridge*
[3]	18.85	12.32	27.24
[4]	16.52	12.56	26.20
[22]	**33.50**	**28.43**	**40.82**
[32]	26.49	12.31	26.50
[17]	-	11.94	31.01
[25]	-	9.55	29.18
Ours	27.17	12.68	33.66

## References

[1] Xiong N., Liu R.W., Liang M., Wu D., Liu Z., Wu H. (2017). Effective alternating direction optimization methods for sparsity-constrained blind image deblurring. Sensors.

[2] Fergus R., Singh B., Hertzmann A., Roweis S.T., Freeman W.T. (2006). Removing camera shake from a single photograph. ACM Trans. Graph..

[3] Shan Q., Jia J., Agarwala A. (2008). High-quality motion deblurring from a single image. ACM Trans. Graph..

[4] Cho S., Lee S. (2009). Fast motion deblurring. ACM Trans. Graph..

[5] Xu L., Jia J. Two-phase kernel estimation for robust motion deblurring. Proceedings of the 11th European Conference on Computer Vision.

[6] Krishnan D., Tay T., Fergus R. Blind deconvolution using a normalized sparsity measure. Proceedings of the IEEE Conference on Computer Vision and Pattern Recognition.

[7] Zhang H., Yang J., Zhang Y., Huang T.S. (2013). Image and video restorations via nonlocal kernel regression. IEEE Trans. Cybern..

[8] Kim T.H., Lee K.M. Segmentation-free dynamic scene deblurring. Proceedings of the IEEE Conference on Computer Vision and Pattern Recognition.

[9] Bi S., Zeng X., Tang X., Qin S., Lai K.W.C. (2016). Compressive video recovery using block match multi-frame motion estimation based on single pixel cameras. Sensors.

[10] Tan F., Liu S., Zeng L., Zeng B. Kernel-free video deblurring via synthesis. Proceedings of the IEEE International Conference on Image Processing.

[11] Šroubek F., Milanfar P. (2012). Robust multichannel blind deconvolution via fast alternating minimization. IEEE Trans. Image Process..

[12] Yuan L., Sun J., Quan L., Shum H.Y. (2007). Image deblurring with blurred/noisy image pairs. ACM Trans. Graph..

[13] Li H., Zhang Y., Sun J., Gong D. Joint motion deblurring with blurred/noisy image pair. Proceedings of the 22nd International Conference on Pattern Recognition.

[14] Tai Y.W., Lin S. Motion-aware noise filtering for deblurring of noisy and blurry images. Proceedings of the IEEE Conference on Computer Vision and Pattern Recognition.

[15] Tai Y.W., Tan P., Brown M.S. (2011). Richardson-lucy deblurring for scenes under a projective motion path. IEEE Trans. Pattern Anal..

[16] Xu Y., Hu X., Peng S. (2015). Blind motion deblurring using optical flow. Optik.

[17] Cho S., Wang J., Lee S. (2012). Video deblurring for hand-held cameras using patch-based synthesis. ACM Trans. Graph..

[18] Zhang H., Yang J. Intra-frame deblurring by leveraging inter-frame camera motion. Proceedings of the IEEE Conference on Computer Vision and Pattern Recognition.

[19] Zhang H., Wipf D., Zhang Y. Multi-image blind deblurring using a coupled adaptive sparse prior. Proceedings of the IEEE Conference on Computer Vision and Pattern Recognition.

[20] Cai J.F., Ji H., Liu C., Shen Z. (2009). Blind motion deblurring using multiple images. J. Comput. Phys..

[21] Takeda H., Milanfar P. (2011). Removing motion blur with space-time processing. IEEE Trans. Image Process..

[22] Chan S.H., Khoshabeh R., Gibson K.B., Gill P.E., Nguyen T.Q. (2011). An augmented Lagrangian method for total variation video restoration. IEEE Trans. Image Process..

[23] Qiao C., Lau R.W.H., Sheng B., Zhang B., Wu E. (2017). Temporal Coherence-based Deblurring Using Nonuniform Motion Optimization. IEEE Trans. Image Process..

[24] Wulff J., Black M.J. Modeling blurred video with layers. Proceedings of the 13th European Conference on Computer Vision.

[25] Kim T.H., Lee K.M. Generalized video deblurring for dynamic scenes. Proceedings of the IEEE Conference on Computer Vision and Pattern Recognition.

[26] Delbracio M., Sapiro G. Burst deblurring: Removing camera shake through fourier burst accumulation. Proceedings of the IEEE Conference on Computer Vision and Pattern Recognition.

[27] Delbracio M., Sapiro G. (2015). Removing camera shake via weighted fourier burst accumulation. IEEE Trans. Image Process..

[28] Delbracio M., Sapiro G. (2015). Hand-held video deblurring via efficient fourier aggregation. IEEE Trans. Comput. Imaging..

[29] Lee D.B., Jeong S.C., Lee Y.G., Song B.C. (2013). Video deblurring method using accurate blur kernel estimation and residual deconvolution based on a blurred-unblurred frame pair. IEEE Trans. Image Process..

[30] Lee D., Heo B.Y., Song B.C. Video deblurring based on bidirectional motion compensation and accurate blur kernel estimation. Proceedings of the 20th IEEE International Conference on Image Processing.

[31] Chan S.H., Nguyen T.Q. (2011). LCD motion blur: Modeling, analysis, and method. IEEE Trans. Image Process..

[32] Gong W., Wang W., Li W., Tang S. Temporal consistency based method for blind video deblurring. Proceedings of the 22nd International Conference on Pattern Recognition.

[33] Zhang L., Zhou L., Huang H. (2017). Bundled kernels for non-uniform blind video deblurring. IEEE Trans. Circuits Syst. Video Technol..

[34] Hassen W., Amiri H. Block matching methods for motion estimation. Proceedings of the 7th IEEE International Conference on E-Learning in Industrial Electronics.

[35] Rublee E., Rabaud V., Konolige K., Bradski G. ORB: An efficient alternative to SIFT or SURF. Proceedings of the 13th IEEE International Conference on Computer Vision.

[36] Song Z., Klette R. Robustness of point feature detection. Proceedings of the 15th International Conference on Computer Analysis of Images and Patterns.

[37] Almeida M.S.C., Almeida L.B. (2010). Blind and semi-blind deblurring of natural images. IEEE Trans. Image Process..

[38] Yang F., Huang Y., Luo Y., Li L., Li H. (2016). Robust Image Restoration for Motion Blur of Image Sensors. Sensors.

[39] Levin A., Weiss Y., Durand F., Freeman W.T. (2011). Understanding blind deconvolution algorithms. IEEE Trans. Pattern Anal. Mach. Intell..

